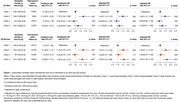# Gender‐Specific Associations Between Social Determinants of Health Clusters and Dementia Risk in Community‐Dwelling Older Adults

**DOI:** 10.1002/alz70860_100599

**Published:** 2025-12-23

**Authors:** Htet Lin Htun, Achamyeleh Teshale, Joanne Ryan, Alice Owen, Trevor T.‐J. Chong, Suzanne Orchard, Anne Murray, Raj C. Shah, Robyn L. Woods, Rosanne Freak‐Poli

**Affiliations:** ^1^ Monash University, Melbourne, VIC, Australia; ^2^ Turner Institute for Brain and Mental Health & School of Psychological Sciences, Monash University, Clayton, VIC, Australia; ^3^ Berman Center for Outcomes and Clinical Research, Hennepin Healthcare Research Institute, Minneapolis, MN, USA; ^4^ Rush University Medical Center, Chicago, IL, USA

## Abstract

**Background:**

Socioeconomic and psychosocial conditions, collectively referred to as social determinants of health (SDH), are interrelated factors influencing dementia risk across the life course. However, the co‐occurrence of adverse SDH is often underexplored. Existing studies often focus on individual SDH or composite indices, potentially overlooking the clustering of multidomain adverse SDH within individuals. This study aimed to identify co‐occurring SDH clusters and examine their associations with dementia risk in community‐dwelling older adults, analysed separately for men and women.

**Method:**

A gender‐disaggregated analysis was conducted among 12,896 community‐dwelling older Australians aged 70+ years without major cognitive impairment at baseline. Latent class analysis identified clusters derived from 72 SDH measures (70 individual‐level and 2 neighbourhood‐level). Cox proportional hazards regression was used to estimate dementia risk over a 12‐year follow‐up (median: 8.4 years), adjusting for age and other known dementia risk factors.

**Result:**

Participants had a mean age of 75.2 years (± 4.3), with 54% women. Four distinct clusters were identified: Class 1 “least disadvantaged” (31.5% men, 30.6% women), Class 2 “most disadvantaged” (20.2% men, 19.4% women), Class 3 “higher social support with Class‐1 features” (22.2% men, 24.1% women), and Class 4 “higher social support with Class‐2 features” (26.1% men, 25.7% women). Compared to Class 1, both men (HR: 1.49, 95% CI: 1.12–1.98) and women (HR: 1.56, 95% CI: 1.17‐2.07) in Class 2, and women in Class 4 (HR: 1.66, 95% CI: 1.28‐2.16) had a significantly greater risk of dementia. No increased risk was observed for men (HR: 0.90, 95% CI: 0.66‐1.23) and women (HR: 1.06, 95% CI: 0.79‐1.43) in Class 3, nor for men in Class 4 (HR: 1.20, 95% CI: 0.91‐1.59) (Figure 1).

**Conclusion:**

Socioeconomic disadvantage was associated with an increased risk of incident dementia. Despite stronger social support, women appeared to be disproportionately affected by adverse SDH in relation to dementia risk. Addressing multidimensional deprivation at the individual, community, and national levels throughout the life course should be central to both new and existing interventions and policies aimed at promoting health equity.